# Immune mechanisms and pathophysiology of T cell-mediated pediatric acute liver failure (TC-PALF)

**DOI:** 10.1097/HC9.0000000000001033

**Published:** 2026-08-20

**Authors:** Jason T.C. Lee, Harry Sutton, Priya Pai, Arianna Barbetta, Shengmei Zhou, Rohit Kohli, Sonya MacParland, Juliet Emamaullee

**Affiliations:** 1 Division of Abdominal Organ Transplantation and Hepatobiliary Surgery, Department of Surgery, Keck School of Medicine, University of Southern California, Los Angeles, California, USA; 2 Division of Gastroenterology, Hepatology and Nutrition, Children’s Hospital Los Angeles, Los Angeles, California, USA; 3 Department of Immunology, University of Toronto, Toronto, Ontario, Canada; 4 Department of Pathology and Laboratory Medicine, Children’'s Hospital Los Angeles, Los Angeles CA, USA; 5 Department of Surgery, University of Rochester, Rochester, New York, USA

**Keywords:** autoimmune hepatitis, liver immunology, pediatric acute liver failure, pediatric liver transplantation

## Abstract

Immune dysregulation in pediatric acute liver failure (PALF) is a distinct phenomenon that has garnered interest with respect to disease outcomes and targeted therapies. Some patients with PALF have an “indeterminate” (iPALF) etiology ranging from acute severe hepatitis to fulminant liver failure. Recent evidence from iPALF demonstrates that a large subset suffers from a unified, immune-mediated disorder. This immune-mediated PALF has been defined by the presence of dense T-cell infiltrates on liver biopsy and is often referred to as T-cell PALF (TC-PALF). TC-PALF has common features with other inflammatory liver diseases, including autoimmune hepatitis, hemophagocytic lymphohistiocytosis, and macrophage activation syndrome, while also demonstrating distinct pathologic features that contribute to liver injury in PALF. In this review, the spectrum of disease, comparisons between young and aged liver immune microenvironments, and the current literature evaluating the immune system during PALF are summarized. Relationships between TC-PALF, other inflammatory liver diseases, and contemporary studies that associate specific immune subsets with this pathology are also reviewed. These studies use precision “omic” technologies to investigate tissue and blood samples in TC-PALF and have opened new lines of investigation into potential genetic, immunologic, and environmental risk factors for disease. Together, recent data suggest that immune dysregulation is a central feature of TC-PALF, and facets of disease offer potential biomarker identification to aid in the clinical management of TC-PALF.

## Graphical abstract

**Figure:**
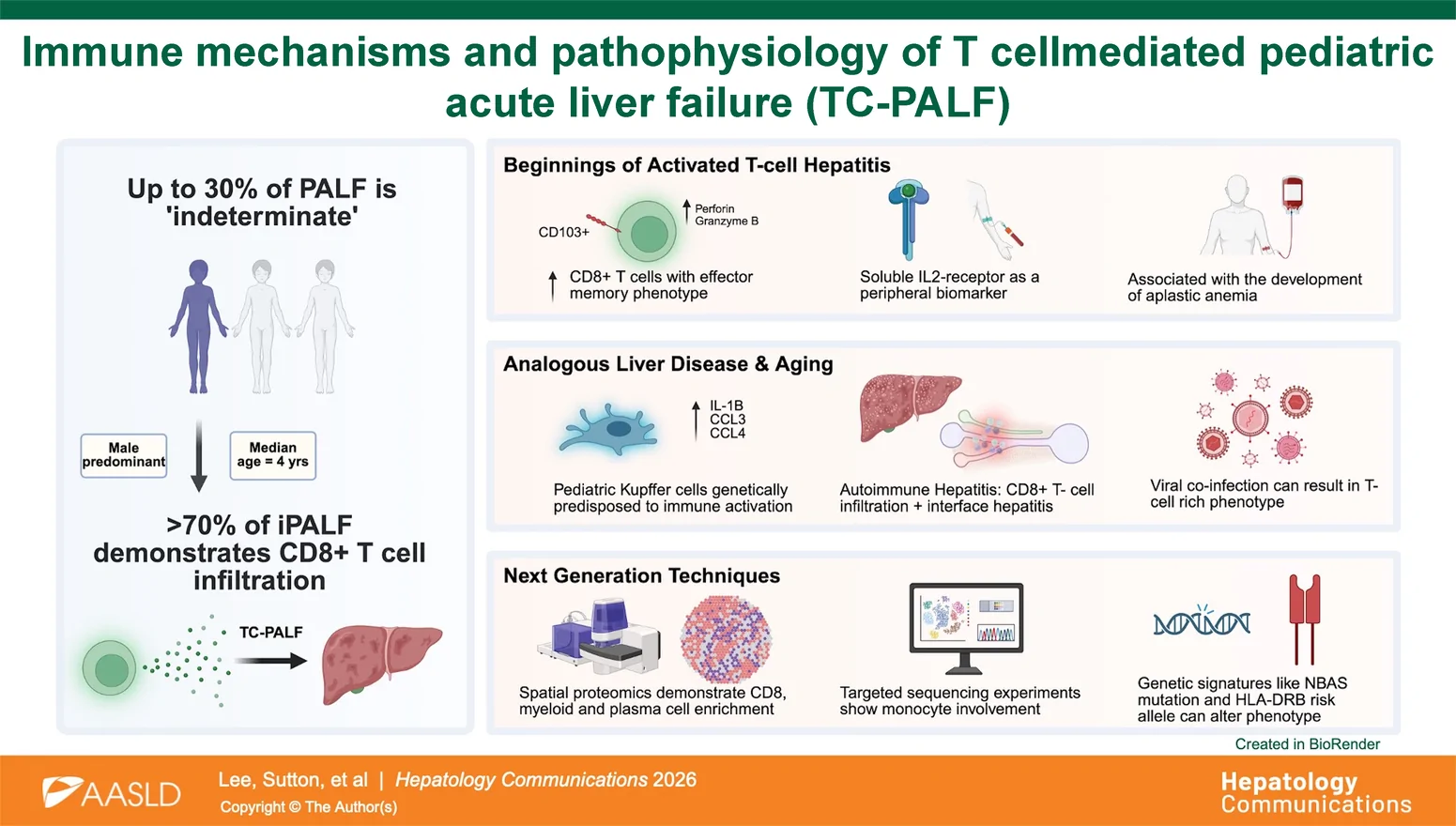

## INTRODUCTION

Pediatric acute liver failure (PALF) is a rapidly progressive, life-threatening condition that causes sudden disease in otherwise healthy children. Although mortality before liver transplantation (LT) approached 70%–95% in early series,^1^ advances in supportive care have improved outcomes, with survival with native liver (SNL) occurring in ~55%–75% of patients today.^2^ Consensus criteria to screen for PALF were established by the Pediatric Acute Liver Failure Study Group (PALFSG), a multinational NIH-funded patient registry established in 1999, as follows: acute disease <8 weeks in duration, refractory coagulopathy (INR >2.0 or 1.5 in the presence of hepatic encephalopathy), and evidence of acute liver damage (through hepatocellular injury or biliary dysfunction).^3^ Common indications for PALF outside of the neonatal period, where genetic causes dominate, include acetaminophen toxicity, metabolic crises, and viral illnesses; however, the plurality of children with PALF go undiagnosed and are given the label “indeterminate.” Over the last 2 decades, diagnostic refinement has decreased the overall percentage of “indeterminate” cases from 49% in the original PALFSG cohort to 12%–30% of patients today, where it is most prominent in toddlers (3 mo to 3 y) at 50%–65% of cases.^4^,^5^,^6^ Indeterminate etiology is uniquely prominent in children, as acute liver failure in adults is more commonly linked to drug toxicities (ie, acetaminophen ingestion) or viral hepatitis, with indeterminate etiology representing <10% of cases in adults.^7^ Thus, indeterminate PALF (iPALF) has remained a diagnostic enigma, prompting numerous studies over the last decade.

There has been a growing body of evidence suggesting that the vast majority (>70%) of iPALF have a hallmark feature of dense intrahepatic CD8+ T-cell infiltration, with associated inflammatory circulatory factors suggestive of immune dysregulation.^8^,^9^,^10^,^11^,^12^ This has led to a novel designation known as activated T-cell hepatitis (TC-Hep or TC-PALF), distinct from other inflammatory liver diseases.^13^ This designation has led to the development of the Treatment for Immune Mediated Pathophysiology (TRIUMPH) study, a National Institute of Health (NIH)-funded, multicenter, randomized controlled trial examining the use of immunosuppressive therapies in the treatment of TC-PALF.^14^ The TRIUMPH intervention study has now closed enrollment, but only preliminary data have been reported to date.^15^

There remains a major gap in our understanding of TC-PALF, including potential inciting factors, patient susceptibility, and the contribution of other important immune subsets within the intrahepatic inflammatory milieu. In this review, we explore potential mechanisms of TC-PALF by summarizing immune-centric studies in the PALFSG and elsewhere (Table 1), highlighting analogous inflammatory liver disease and unique aspects of pediatric immunity that may contribute to age-associated findings, and providing an overview of recent discoveries made with advanced investigative techniques. Further, different forms of inflammatory pediatric hepatitis are compared with disease mechanisms uniquely attributed to TC-PALF.

**TABLE 1:** Summary of experimental studies investigating immune contributors to inflammatory liver disease

Study			Cell type									
iPALF, TC-PALF, ASH	Population	Technique	Cytotoxic (CD8) T cells	Helper (CD4) T cells	T regs	NKT cells	NK cells	B cells	Plasma cells	Monocyte/macrophages	Dendritic cells	Summary
Bucuvalas J (2013) JPGN^8^	PALFSG - SNL (51) - LT (21) - Death (5)	Flow cytometry	●	x	x	◯	◐	x	x	◯	x	- Serum sIL2Ra differentiated patient outcomes
Chapin CA (2018) Hepatology^11^	Lurie Children’s (Chicago) - iPALF (33) - dPALF (14) - AIH (9)	IHC TCR-seq	●	◯	x	x	◯	◯	x	●	x	- Dense hepatic infiltrates of CD8+CD103+ (resident memory) T-cells in iPALF vs. dPALF - TCR-seq has increased clonality in iPALF vs. dPALF
Chapin CA (2020) JPGN^12^	PALFSG - iPALF (37) - dPALF (18)	IHC TCR-seq	●	◯	x	x	x	◯	x	◐	x	- Dense CD8+CD103+perforin+ staining in iPALF vs. dPALF - TCR-seq has increased clonality in iPALF vs. dPALF
Chapin CA (2021) Hep Comm^16^	Lurie Children’s (Chicago) - iPALF (17) - dPALF (6)	IHC MesoScale RNAseq	●	◐	◐	x	x	x	◯	◐	◐	- iPALF had dense CD8 staining with Th1 immune analytes - Two transcriptional PALF phenotypes, group 1 associated with iPALF and dense CD8 staining - Group 1 linked to inflammation, T-cell activation, antigen stimulation
Leonis MA (2021) Hepatology^17^	PALFSG - 94 PALF patients	Immulite flow cytometry	●	◯	x	x	◯	x	x	◯	x	- 4/12 immune activation markers (IAMs) associated with CD8+ T-cells distinguished low-high cohorts - High IAM cohort linked to iPALF and higher disease severity
Chapin CA (2023) PLoS One^9^	Lurie Children’s (Chicago) - TC-PALF (4) - iPALF (4)	Flow cytometry	●	◐	x	x	◯	◯	◯	◯	x	- TC-PALF had higher cirulating CD8+ Tem cells vs. other iPALF - Cells more likely to be CD103+ (Trm phenotype)
Lee J (2025) Hepatology (AASLD)*^18^	CHLA (Los Angeles) - Healthy (33) - PALF (66) - iPALF (34) - SNL (24)	Imaging mass cytometry Plasma proteomics	●	●	●	x	●	●	◯	●	◯	- Exhausted CD8 T-cells and macrophages enriched in PALF - PD-L1+ macrophages/hepatocytes to CD8+ T-cells interactions predict SNL
Nguyen TH (2025) J Allergy Clin Immunol^19^	US Multicenter - Healthy (15) - iSH (14) - PALF (7) - HLH (5)	Flow cytometry Cytokine profiling	●	●	x	x	x	x	x	●	x	- CD8 T-cell activation predictive of iSH progression to iPALF - sILR2/ferritin ratio distinguishes iSH from PALF and HLH
Sutton H (2025) Hepatology (AASLD)*^20^	Toronto - iPALF (8) - ALF (6) - dPALF (3) - Healthy (3)	scRNA-seq TCR-seq	●	◯	◯	x	◯	◯	x	◯	x	- Cytotoxic T-cells in iPALF express NK marker NKG2D and lacked canonical TCR activation marker (CD25) - Clonality experiments found polyclonal population, interactions via CCL5:CCR5 signaling
Diamond T(2026) Hep Comm^15^	PALFSG - TC-PALF (16) - Other PALF (55) - Healthy (15)	Flow cytometry scRNA-seq Olink proteomics	●	●	x	x	●	●	◯	●	◯	- Two endotypes from CD8 and perforin expression in blood/liver in TC-PALF - High perforin endotype associated with CD8 Trm signature in blood - Low perforin endotype associated with aberrant monocyte response and class-switched B-cells
Viral												
Chen Z (2018) PNAS^21^	Liver Transplant Center (Cagliari, Italy) - Adult HBV ALF (4)	Microarray Immunohistochemistry	◯	x	x	x	x	●	x	◯	x	- HBV ALF is a B-cell dominant disease with IgM/IgG targeting of HBcAg - Mechanism for liver injury independent of T-cells
Kim J (2018) Immunity^22^	Chung-Ang University (Seoul) - AHA (76)	Flow cytometry Cytokine profiling IHC	●	x	x	x	x	x	x	●	x	- Hepatitis A induces TCR-independent activation of CD8 T-cells - T-cells exhibit innate cytotoxicity through NKG2D and NKp30 - Liver injury correlated to the degree of innate cytotoxicity
Morfopoulou S (2023) Nature^23^	UK Collaborative - Viral (38) - Healthy (66) - Immunocompromised (21)	Viral metagenomics RNAseq Proteomics	●	x	x	x	x	●	x	x	x	- High AAV2 DNA in 27/28 cases, some HHV-6B and HAdV - Histologic enrichment of T and B cells in liver - Enrichment of class II HLA, complement, and immunoglobulin variable regions in cases, viral trigger of immune pathways
Ho A (2023) Nature^24^	NHS Glasgow Biorepository - Viral (32)	Viral metagenomics IHC PhenoCycler	x	●	x	x	x	x	x	●	x	- AAV2 detected in 26/32 cases - Prominent T-cell signature in liver, linked to CD4 T-cells and class II HLA with identified protective alleles
Servellita V (2023) Nature^25^	US Multicenter - HAdV cases (16) - Healthy (113)	Viral metagenomics	x	x	x	x	x	x	x	x	x	- 13/14 cases positive for AAV2 in blood - Coinfection with HAdV, EBV, HHV6, and enterovirus enriched in cases vs controls
de Kleine RH (2025) mBio^26^	Dutch National Center - Hep A-E neg PALF (5) - Controls (6)	Viral metagenomics RNAseq Proteomics	x	x	x	x	x	x	x	●	x	- AAV2, adenovirus, and herpes found in explant liver of cases - Liver explant cases associated with monocyte activation signatures
Röttele F (2025) Gut^27^	European cohort - AHUO (12)	Viral PCR IMC	●	◯	◯	x	x	◐	●	●	x	10/12 patients had previous +COVID with CoV2 antigen expression in 11/12, high CD8 infiltration and peripheral activation linked to the most severe hepatitis, interactions between CX3CR1+ endothelial and myeloid populations with CD8s forming a triad

*Note:* The table highlights patient populations included, investigatory techniques, cell types analyzed, and the author's conclusions. Pre-peer-reviewed evidence published in non-manuscript form (such as meeting abstracts) is indicated by an asterisk (*). Cell type contribution to disease is marked as follows: cell types that significantly differentiate grouping (●), cell types that trend toward group differentiating (◐), cell types that are examined but do not distinguish (◯), and cell types not assessed in the study (x).

AHA, acute hepatitis A; AHUO, acute hepatitis of unknown origin; AIH, autoimmune hepatitis; AAV, adeno-associated virus; dPALF, diagnosed pediatric acute liver failure; EBV, Epstein–Barr virus; hAdV, human adenovirus; HBV, hepatitis B virus; HHV, human herpesvirus; HLH, hemophagocytic lymphohistiocytosis; IHC, immunohistochemistry; IMC, imaging mass cytometry; iPALF, indeterminate pediatric acute liver failure; ISH, indeterminate severe hepatitis; JPGN, Journal of Pediatric Gastroenterology; LT, liver transplant; PALFSG, Pediatric Acute Liver Failure Study Group; SNL, survival with native liver; TCR-seq, T-cell receptor sequencing; TC-PALF, T-cell-mediated pediatric acute liver failure.

## THE BEGINNINGS OF ACTIVATED T-CELL HEPATITIS: WHAT DO WE KNOW?

### The T-cell story—Results from the pediatric acute liver failure study group

Initial literature documenting histologic hepatocyte loss and inflammatory parenchymal infiltrate began with early single-center studies in the 1980s.^28^ The hypothesis of immune dysregulation was corroborated by early findings of NK-cell dysfunction and circulatory cytokine profiles akin to systemic inflammatory response syndrome (SIRS), resulting in a concerted effort to investigate mechanisms of disease following the establishment of the PALFSG in 1999.^4^,^8^ Most available mechanistic data in the TC-PALF space have relied on the multicenter cohort established by the PALFSG thus far.

In a flow cytometric study of 77 patients with PALF (~35% iPALF) investigating numerous T-cell and NK-cell-related circulatory biomarkers, deleterious outcomes (LT or death) were most associated with increased soluble interleukin 2 receptor alpha (IL2Rα) and CD8+ cells, respectively.^8^ These findings were corroborated in a subsequent multicenter study, which identified the dense enrichment of CD8+, CD103+, and perforin+ immunohistochemical staining in iPALF patients (n=33) as compared with autoimmune hepatitis (AIH) (n=9) or their determinate PALF (dPALF) counterparts (n=14).^11^ Further investigation revealed these CD8^+^CD103^+^ T-cells were CD45RA^−^CCR7^−^, indicative of a tissue-resident, effector memory T-cell phenotype.^11^ T-cell receptor sequencing to evaluate clonality determined that iPALF is associated with a higher proportion of clonal T cells at 17.5% across the top 20 TCR-β sequences, compared with 6.5% and 7.4% for AIH and dPALF, respectively.^11^ When evaluating other immune cell types in PALF tissue, macrophages (CD163) were expanded in iPALF while B-cells (CD20), NK-cells (CD56), and CD4+ T-cells were not.^11^ These results were corroborated by the PALFSG registry cohort of 37 iPALF and 18 dPALF patients, with a significant enrichment of CD8, perforin, CD103, and TCR clonality in iPALF tissue.^12^

Building on their previous work, Chapin et al^16^ further investigated the inflammatory milieu of iPALF through the quantification of 39 cytokines and bulk RNAseq in liver tissue from 17 iPALF and 6 dPALF patients at a single center.^16^ Of those patients who exhibited dense CD8+ T-cell staining, CXCL10, CXCL12, and IL-16 were the analytes most associated with this histology.^16^ Transcriptional analysis coupled with unsupervised clustering identified 2 groups, one that was associated with an inflammatory signature associated with iPALF patients whose tissue demonstrated dense CD8 staining. Examples of genetic themes associated with this group include chemotaxis (*CCR5*, *CXCR6*), immunomodulation (*TIGIT*, *CTLA4*, *LAG3*), and the class I major histocompatibility complex (*HLA-A*, *HLA-B*, *HLA-F*). These findings are corroborated by an adjacent study in the PALFSG cohort of 47 PALF patients looking at immune activation markers (IAMs) to qualify immune dysregulation.^17^ Factors associated with perforin, granzyme, sIL2R, and CD8 T-cells were associated with gradations of an IAM profile that separated cases based on severity and were preferentially linked to iPALF patients.^17^ Taken together, these studies rationalize further investigation of T-cell-driven mechanisms and adoption of the TC-PALF nomenclature.

Initial reports from PALFSG and the TRIUMPH study were published, looking at 16 TC-PALF patients compared with dPALF patients (n=55), patients with aplastic anemia, and healthy controls (n=15).^15^ Patients with TC-PALF were stratified secondary to perforin staining within the liver and circulating CD8+ T-cell profiles, with high (n=11) and normal (n=5) perforin “endotypes” defined by differential flow cytometric, transcriptomic, and circulatory proteomic signatures. Circulatory cells in TC-PALF had a unique CD8^+^CCR7^lo^CD45RA^lo^HLA-DR^+^CD103^+^ transcriptional signature reminiscent of effector memory T-cells within the hepatic parenchyma. The TC-PALF “high perforin” endotype was marked by greater cytolytic and IFNγ-associated activity, with supporting resident memory T-cell expression of *ITGAE*, *CD38*, *PRDM1*, and *RUNX3*. Re-clustered cells within this endotype that were non-resident memory phenotype had gene ontology terms centered around TCR signaling. Other potentially involved immune cell types were explored, including B-cells, with evidence of restricted class switching due to high *IGHM* and *IGHD* expression as compared with *IGHG*. The use of contemporary in silico predictive interaction tools suggested interactions between CD8+ T-cells, NK cells, and circulatory monocytes that corroborate tissue-based macrophage populations observed within the original PALFSG studies^11^,^12^ and those associated with viral infections.^23^,^24^,^25^ Taken together, this work suggests an interferon-mediated, inflammatory process mediating immune responses in TC-PALF.

## iPALF IS A UNIQUELY PEDIATRIC DISEASE: DOES THE LIVER MICROENVIRONMENT CHANGE WITH AGING?

There is a growing appreciation for age-related influence on liver and liver-resident immune system function. The prominence of TC-PALF in the pediatric population, in contrast to its near absence in adults, further suggests that age-specific structural or functional differences within the pediatric immune microenvironment may contribute to its underlying pathology.^5^ Although select adult cohorts, such as those undergoing hematopoietic stem cell transplant, can experience aplastic anemia-associated hepatitis similar to TC-PALF,^29^ other pediatric-specific liver diseases, such as biliary atresia and gestational alloimmune liver disease, demonstrate unique rapid fibrotic and cirrhotic features that highlight unique features in young liver resident cellular function. Most of the published liver immune microenvironment literature focuses on non-pediatric populations, that is, adult or “aged” patients. Therefore, one can infer the characteristics of a “young” liver immune microenvironment in the context of these established studies, although many of the proposed mechanisms remain speculative.

### The immune system in the aging liver and its implications for the liver immune microenvironment in children

Two concepts are prominent in liver aging literature, namely inflammaging and immunosenescence; these are phenomena that refer to the structural and phenotypic changes associated with hepatic architecture and function over time.^30^ Throughout life, accumulation of transient, low-grade inflammation events causes pseudocapillarization, increasing vascular thickness, scar deposition, and decreasing fenestration of liver sinusoidal endothelial cells (LSECs) that control the parenchymal-circulatory interface.^30^ Conversely, at the beginning of life, livers are structurally primed for interactions between circulating immune cells and parenchyma, leading to unique interacting partners and potential infectious triggers for disease due to enterohepatic circulation.^31^ Aging is associated with a shift toward innate immune cell enrichment (NK and macrophages) away from adaptive (T-cells and B-cells).^30^ Although both M1 and M2 macrophage accumulation have been implicated in aging livers, their capacity to participate in normal function, such as phagocytosis, is diminished, skewing towards fibrosis rather than regeneration.^30^

This effect is replicated across both the innate and adaptive immune system, where the capacity to mount robust responses to infectious stimuli is diminished in aging due to naïve T-cell deficiency secondary to thymic involution and accrual of exhaustive T-cell phenotypes expressing factors such as *Pdcd1*, *Ctla4*, *Lag3*, and *Tigit*.^32^ Contemporary PBMC studies inclusive of healthy patients as young as 2 months corroborate some of these findings, with a prominence of naïve T-cells, CD16+ monocytes, plasmacytoid dendritic cells, as well as constitutive activation of interferon-related gene expression.^33^ Interestingly, mitigation of age-related immune changes can be pursued through senolysis, caloric restriction, and exercise.^30^ Induced reconstitution of the young immune system through transient signaling via the Notch, FLT3L, and IL-7 signaling pathways can promote the expansion of previously lost common lymphoid progenitors, restore thymopoiesis, and boost anti-tumor response through enhanced CD8 T-cell function.^32^ Taken together, these results suggest that the pediatric immune system retains the capacity to robustly mount adaptive immune responses due to the lack of accumulation of age-related deleterious changes.

### The liver immune microenvironment in children—-Priming for T-cell action?

A recent study established an atlas of pediatric liver cells and compared their transcriptomic signatures to those of the adult liver.^34^ Tissue from 9 pediatric donors was compared with 7 adults, with 35 distinct populations identified.^34^ Although somewhat contradictory to previous studies noting increased IL-1β expression in aged macrophages,^30^ this atlas identified genes associated with immune activation within the Kupffer-like cells in children, including *CCL3*, *CCL4*, and *IL1B*.^34^ Enhancement of IL-1β expression was induced further through stimulation by lipopolysaccharide (LPS), noting the increased sensitivity of pediatric cells as compared with their adult counterparts.^34^ This heightened inflammatory signaling is speculated to drive increased T-cell and monocyte recruitment within young livers and may explain why the T-cell response is much more dominant in TC-PALF as compared with inflammatory liver diseases in adults.^34^

## DRAWING PARALLELS BETWEEN TC-PALF AND ANALOGOUS INFLAMMATORY LIVER DISEASES

After considering features related to the underlying liver architecture and immune homeostasis in the “young” liver, understanding how TC-PALF relates to other forms of inflammatory liver disease is crucial. Specifically, understanding how hepatic parenchyma is damaged by inflammatory processes and how the injured liver mediates regeneration during analogous inflammatory liver diseases provides important context for understanding features of immune dysregulation in TC-PALF.

### Autoimmune hepatitis and associated systemic inflammatory conditions

**FIGURE 1:**
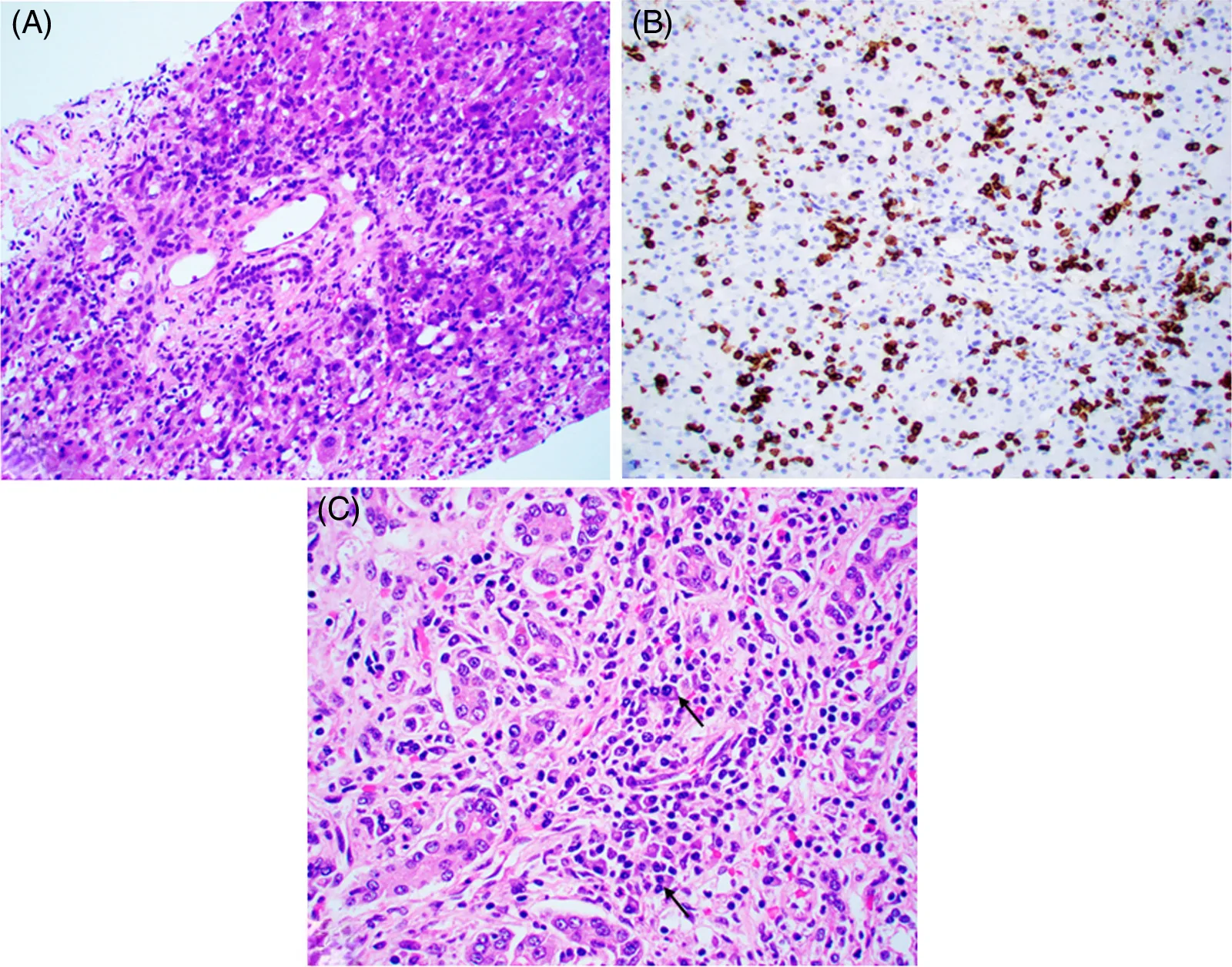
Representative histopathologic features of activated T-cell-mediated pediatric acute liver failure (TC-PALF) and autoimmune hepatitis (AIH). (A–B) Representative liver tissue sections from a patient with TC-PALF. (A) H&E-stained section shows prominent lymphocytic inflammation; the central portal tract is relatively spared. (B) Immunohistochemical staining for CD8 highlights a CD8^+^ T-cell infiltrate. (C) H&E-stained section from a patient with AIH demonstrates portal and periportal inflammation with numerous plasma cells (arrows) and interface hepatitis. Abbreviations: AIH, autoimmune hepatitis; H&E, hematoxylin and eosin; TC-PALF, T-cell-mediated pediatric acute liver failure.

**FIGURE 2:**
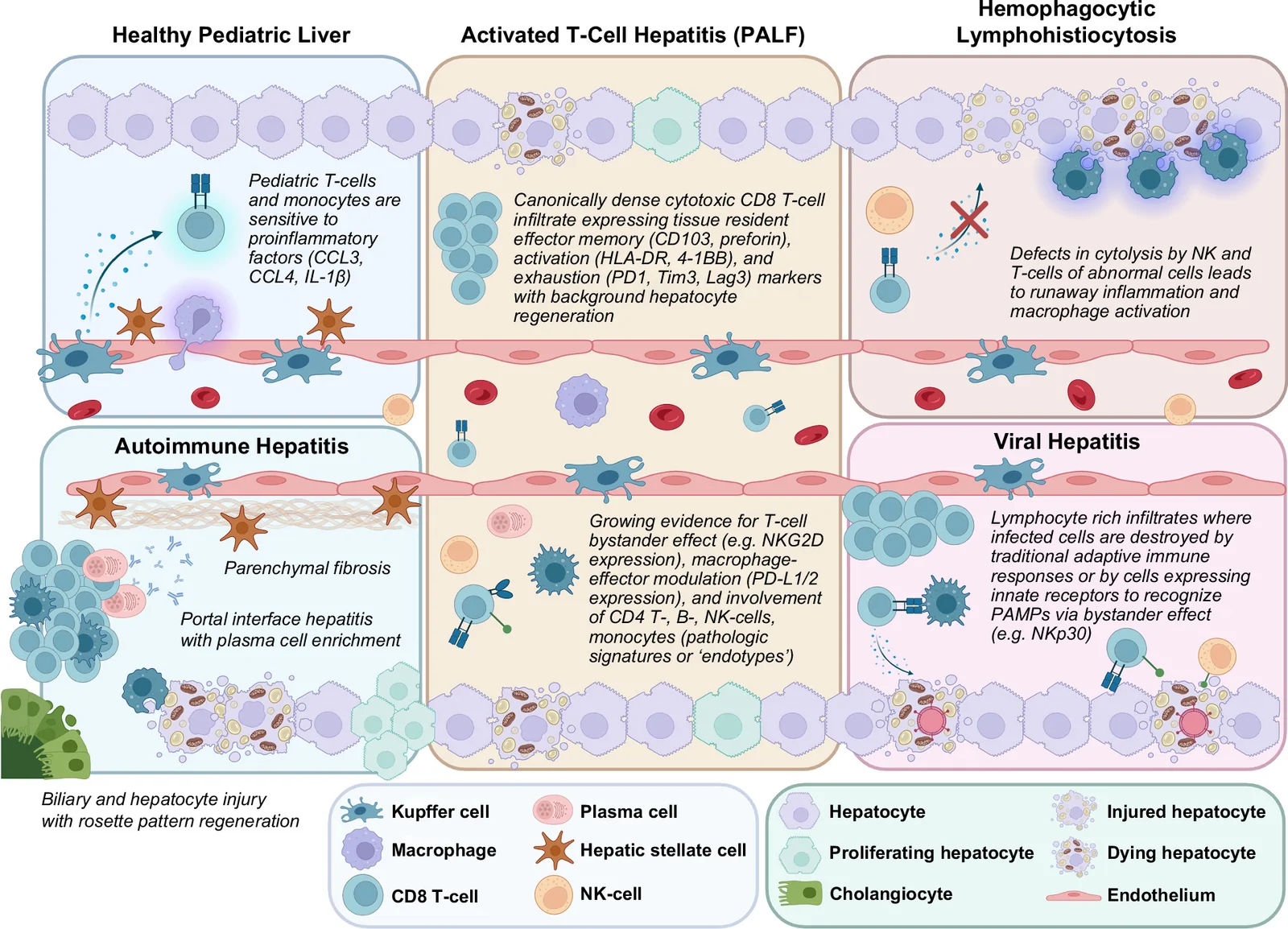
Proposed immunologic players and histologic patterns in healthy pediatric liver, activated T-cell hepatitis, autoimmune hepatitis, viral hepatitis, and hemophagocytic lymphohistiocytosis. Graphical depiction of current literature regarding the immune players driving inflammatory liver disease. Abbreviations: NK, natural killer; PALF, pediatric acute liver failure; PAMPs, pathogen-associated molecular patterns.

Autoimmune hepatitis (AIH) is considered a distinct inflammatory liver disease from TC-PALF. Distinguishing features of AIH from PALF include: a varied acute-to-chronic timeline, presence of circulating autoantibodies (eg, ANA, anti-SMA, anti-LKM-1—although seronegative AIH is possible), and distinct histologic features (eg, mixed lymphoplasmacytic infiltrates from the portal tracts, termed “interface hepatitis,” fibrosis, and islands of parenchymal regeneration) as depicted in Figure 1.^35^ As with most autoimmune diseases, AIH has a female predominance with a median age of presentation at 10–12 years old.^35^ This contrasts with TC-PALF, which occurs earlier at a median age of 4, exhibits a slight male predominance,^12^,^13^,^15^ lacks canonical AIH histologic features, and is instead replaced by a dense cytotoxic T-cell intrahepatic infiltrate.^4^,^11^ These are not “hard and fast rules”; however, where features can be shared across several different conditions (ie, “interface hepatitis” can be present in viral hepatitis).^36^ These observations suggest that hepatocyte-destructive inflammatory liver disease falls on a continuum, and there are likely universal mechanisms guided by the resident immune architecture that ultimately dictate its course and progression (Figure 2).

Parallels between the liver injury pattern in TC-PALF exist when compared with other systemic inflammatory diseases, such as hemophagocytic lymphohistiocytosis (HLH) and macrophage activation syndrome (MAS).^37^ Although the literature to this point suggests a clonal cytotoxic T-cell population infiltrating the liver tissue, links to familial HLH are particularly interesting due to the underlying mechanisms of HLH, where a patient’s immune system has cytolytic defects in the setting of T and macrophage hyperactivation.^37^ This inability to perform targeted cell killing is associated with a delay in terminating the inflammatory phase of an injury stimulus, thereby leading to persistent elevation of interferons such as IFNγ in the setting of a DAMP/PAMP milieu.^19^ These are contrasted with features that distinguish TC-PALF, such as CD103 and perforin enrichment in resident memory T-cells.^15^,^19^ Drawing parallels to observations seen in acute hepatitis A infection, memory CD8+ T-cells can adopt innate-like cytotoxicity with the expression of NKG2D and NKp30 activating receptors and damage hepatic parenchyma in a TCR-autonomous pathway known as “bystander activation.”^22^ Recently, a similar NKG2D+ T-cell population was identified in the intrahepatic lymphocytes of children with TC-PALF, suggesting bystander activation may contribute to disease pathogenesis.^20^ HLH, viral hepatitis, and TC-PALF demonstrate IFN-γ prominence, where this common feature may represent a shared checkpoint that ultimately causes inflammation-mediated hepatic damage.

### Expanding iPALF triggers—Āre viruses the mystery cause?

There is a spectrum of hepatitis severity under the umbrella of “PALF,” ranging from acute severe hepatitis (ASH) to fulminant hepatic failure.^38^,^39^ The discrepancy in nomenclature reflects the severity of the disease and the degree of synthetic liver dysfunction. The “acute severe hepatitis” label is particularly prominent within the spike in cases worldwide in 2022, coincident with the COVID-19 pandemic, with a relatively low transplant rate (~9%) and no definitive etiology, although studies speculate the involvement of human adeno- (HAdV) and adeno-associated viruses (AAV).^40^ These cases may inform us of complementary or early-stage pathways that cause injury across the gamut of inflammatory liver disease.

In March 2023, 3 concurrent cohort studies were published in *Nature* investigating children with unexplained PALF in 2022 from Scotland, England, and the United States, coincident with the COVID-19 pandemic.^23^,^24^,^25^ Despite the timing, COVID-19 did not appear to be responsible for the outbreak of ASH cases. Before this, viral metagenomic testing in the PALFSG was largely equivocal, with 20% of PALF patients testing positive for various non-hepatitis viruses (HSV, EBV, CMV, HHV6, adenovirus, and parvovirus).^41^ Spurred by initial observations that HAdV, especially HAdV-41, was implicated in ASH, multiple studies began to examine AAV as a potential cause of disease.^42^ All 3 groups reported high rates of AAV2 in children with unexplained ASH.^23^,^24^,^25^

AAVs are largely considered non-pathogenic and are ubiquitously present in humans.^23^ They are classified as “dependoparvoviruses,” requiring coinfection with a secondary virus for intracellular replication.^23^ All three cohort studies performed PCR sequencing on a combination of stool, whole blood, plasma, and liver tissue, identifying a total of 96%, 81%, and 93% of cases that were positive for AAV2 in acute hepatitis patients as compared with 9.2%, 7%, and 3.5% of control cases in London, Scotland, and the United States, respectively.^23^,^24^,^25^ All patients tested positive for a coincident virus (most commonly adenovirus and human herpesvirus 6B (HHV-6B), with 100% positivity for HHV-6B in the explanted livers of the London cohort and not in any patients who SNL.^23^ These findings suggested the possibility of multiple disease triggers, where disease severity can increase secondary to simultaneous factors. Although limited to select samples, immunohistochemical data in these studies revealed variable CD8 staining in some of the reported patient cohorts.^43^

Patient intrinsic risk factors, such as polymorphisms in human leukocyte antigen (HLA), can also influence disease severity through immunomodulation. HLAs are the human-specific homolog of the major histocompatibility complex (MHC) responsible for antigen presentation to effector and helper T-cells, where allelic variation can significantly influence facets of adaptive immunity. Researchers in Scotland performed HLA typing of 27 patients with acute hepatitis and compared their findings with 67 local donor control samples.^24^ The authors reported that 93% of patients were positive for at least one copy of the MHC class II HLA-DRB1*04:01 risk allele, compared with 15% of healthy controls; increased positivity for HLA-DQA1*03:03 was also observed in patients with PALF.^24^ This is an intriguing finding, as MHC class II is canonically expressed on antigen-presenting cells to activate CD4+ T-cells. These findings were corroborated in their study by significant immunostaining for CD4, CD8, CD68, CD20, and MX1 in liver tissue from an AAV2 patient using PhenoCycler/CODEX spatial proteomics, consistent with infiltration of T-cells, macrophages, and B-cells, and activation of the innate immune system.^24^ In addition to the prominence of cytotoxic CD8 T-cells tied to MHC class I in traditional viral responses, the authors speculate the involvement of CD4+ T-helper cells as playing a contributory role in these cases. In response to these HLA typing findings, a group from Japan reported HLA typing of 25 indeterminate PALF cases from 2000 to 2021 and identified protective associations of class I MHC (associated with cytotoxic CD8+ T-cells) alleles HLA-B*52:01 and HLA-C*12:02 in their patients, as evidenced by their complete absence in affected patients.^44^ While these findings require further validation, they highlight a potential role for individual immunophenotypes as a risk factor for iPALF and as a contributor to its pathogenesis.

### A potential role for AAV in iPALF—Do AAVs drive liver injury in general?

The relationship between AAV and liver dysfunction has received renewed interest after several deaths due to ALF in adult and pediatric patients receiving AAV vector gene therapy.^45^ Acute severe hepatitis, with and without acute liver failure, is now a well-recognized side-effect of AAV vector gene therapies. Over a dozen AAV vector gene therapies, utilizing several viral serotypes, are currently available or undergoing clinical trials for a variety of genetic disorders, including Duchenne muscular dystrophy, small muscle atrophy, hemophilia A and B, and X-linked myotubular myopathy.^46^ Hepatotoxicity is extremely common in patients receiving AAV vector therapy, and rates may be as high as 80%–90%, even with the use of prophylactic corticosteroids.^47^ Despite the high rates of hepatitis, liver function is rarely affected beyond the acute exposure, and most patients are managed with outpatient monitoring and various immunosuppressive agents.^48^

The mechanism of liver injury induced by AAV vector therapy is poorly understood, and its relationship to the pediatric cases described in the *Nature* series is unknown.^23^,^24^,^25^ Whether the liver inflammation in patients receiving AAV vector gene therapy is a result of direct cytotoxic effects of viral proteins or a dysregulated immune response, or some combination of the 2 remains to be determined. Both the viral capsid and the transgene being carried by the AAV vector can induce immune responses. Pre-clinical studies from both mice and non-human primates demonstrate dense T-cell infiltration in the liver tissue of AAV vector therapy recipients. A single histologic case study of a patient receiving AAV vector therapy exhibited notable similarities to TC-PALF, including CD8 positivity on IHC staining.^49^ Whether these are expanded, pre-formed memory T cells activated through MHC class I presentation of viral epitopes, or a non-specific polyclonal T-cell response remains unknown. It has also been proposed that features of the innate immune system, namely Toll-like receptor 9 (TLR9), may detect unmethylated cytidine-phosphate guanosine sequences in the transgene DNA and induce an immune response.^50^ This is contrasted by evidence in 2 patients (ages 15, 16) who were administered an AAV vector-based therapeutic (Delandistrogene Moxeparvovec) for Duchenne muscular dystrophy, who experienced ALF with no appreciable CD8 T-cell infiltration on biopsy.^51^ Thus, the weight of patient-specific factors, such as atopy, age, and immune function in evaluation and further monitoring of AAVs in the pathogenesis of iPALF remains an important area of study.

## IMMUNE DYSREGULATION IN iPALF: RECENT INSIGHTS FROM “OMIC” STUDIES

In the absence of an established pre-clinical model of TC-PALF or PALF secondary to AAV exposure, researchers must rely on human tissue samples to study disease mechanisms. The rarity of these conditions makes large cohort studies challenging and limits the interpretability of the findings. Several murine models of ALF have been developed to replicate various human diseases, and concanavalin A (ConA) has previously been used as a surrogate for immune-mediated liver disease.^52^ However, the murine immune response to ConA is unlikely to reflect the immune-mediated liver injury in humans. A true recapitulation of the human immune response in mice may be unachievable, and techniques such as organoids might provide a better platform to study these rare diseases.

### Spatial proteomic techniques

The advent of cutting-edge single-cell sequencing and spatially resolved studies has greatly increased the depth to which independent patients can be characterized. One of the more sophisticated spatial analyses of indeterminate causes of ASH was conducted in Europe and attributes liver sequelae to COVID-19, contrary to the previously discussed studies.^27^ In their multicenter cohort of 12 children with unexplained ASH, defined as ALT>500 IU/L, a 40-plex imaging mass cytometry panel was used to create a spatially resolved, single-cell proteomic map of the affected liver samples. Portal inflammation and interface hepatitis were present in most cases, although other canonical features of AIH (plasma cell infiltration, bile duct lymphocytosis) were not observed. Within their patient cohort, “immune high” (8/12 patients) and “immune low” (4/12 patients) groups were classified based on immune cell density >1750 cells/mm^2^. These “immune high” patients demonstrated enrichment in CD45+ leukocytes, CD8+ T-cells, myeloid cells, and plasma cells (with marginal significance for CD4+ T-cells, B-cells, and regulatory T-cells) when compared with “immune low” counterparts. Intrahepatic and peripheral immune cells were isolated and compared, noting a pattern consistent with intrahepatic and peripheral T-cell activation. Intrahepatic CD8+ T-cells expressed both activation and exhaustion markers (CD38, PD-1) as well as markers linked to a resident memory phenotype (CD103+, CD69+, CXCR6+) and consistent with cells previously identified in the PALFSG literature.^27^

The strength of spatial proteomics lies in its preservation of in situ spatial relationships between the entire inflammatory milieu. Tissue from “immune high” patients was enriched in interactions between activated (TIM-3+, PD-1+) or effector-like (Granzyme B+, CX3CR1+) CD8+ T-cells and M2-skewed (CD163+, CD204+, TIM-3+) myeloid cells. Construction of cellular neighborhoods corroborated this finding, with injury-associated cellular regions enriched with CX3CR1+CD8+ T cells, LSECs, and M2-skewed myeloid cells. This finding was speculated to be a pathological cellular triad within the inflamed liver with close proximity of detectable SARS-CoV-2 antigens.^27^ Importantly, this study demonstrates the feasibility of identifying patient outcomes based on intrahepatic cellular identity and identifying patterns within the tissue’s inherent spatial programming. Emerging studies in PALF also corroborate this spatial programming, where a single-center cohort of 66 patients with PALF (34 with iPALF) demonstrates significant enrichment of intrahepatic CD8 T-cells coincident with exhaustive markers and clustering around immunoregulatory ligand (PD-L1+) expressing populations (ie, hepatocytes and macrophages) and neighborhoods.^18^ These neighborhoods in turn were also predictive of SNL, thereby underscoring the importance of spatial resolution in future investigatory studies.

### Metagenomic, transcriptomic, and bulk proteomic techniques in PALF

In response to the cohorts of PALF cases associated with AAV2 worldwide, a group of Dutch researchers sought to utilize sequencing technologies to investigate the mechanisms of PALF in their cohort of 15 patients as compared with 6 healthy controls.^26^ The authors utilized a combination of targeted transcriptomic and viral metagenomic sequencing to identify potential viral triggers of PALF and to characterize the intrahepatic inflammatory milieu. Nine viruses were examined (AAV2, HAdVC, HAdVF-40/41, EBV, HHV6, HHV7, VZV, TTV, and HPyV), with AAV2 demonstrating the most robust presence in the plasma and liver of 2/5 cases. These 2 AAV2 patients possessed the HLA-DRB1*04:01 MHC II risk allele, the same haplotype seen in the Scottish cohort.^24^ These viruses were not mutually exclusive to PALF cases, with several of the healthy controls also positive within the liver tissue.^26^

Targeted transcriptomic sequencing was then pursued with a >600 gene panel largely focused on inflammatory factors. Transcriptomic signatures readily delineated PALF from healthy individuals through machine learning approaches. Gene set enrichment analysis (GSEA) was significant for enrichment of pathways associated with “regulation of antigen presentation,” “monocytes,” and “chemokine clusters,” with a reduction in “complement” in PALF over controls, contrasting with initial studies that prioritize T-cell-centric signatures. Interestingly, this assay was sensitive enough to detect distinct pathways attributable to corticosteroid treatment in one PALF case that lacked many of the prominent pro-inflammatory pathways identified in others.^49^ External validation was performed by comparing transcripts between the ASH cohort from England^23^ and a small 4-patient Italian cohort of hepatitis B (HBV) associated ALF.^21^ Based on machine learning, the 2 ASH cohorts of unknown origin were grouped together compared with healthy controls and HBV-associated PALF. GSEA on all patients was once again prominent for the involvement of monocytes and highlighted adjunctive immune contributors in PALF.

These findings were corroborated with a focused 92 protein panel using Olink proteomics on 3 PALF cases and 4 healthy controls. Of 61 detectable proteins, 27 were directly linked to the previously quantified transcripts, with 16 demonstrating high correlation with expression (Pearson correlation ρ > 0.5), largely linked again to chemokines and cytokines associated with macrophage and T-cell activation.^26^ Taken together, these data highlight the prominence of monocytes and macrophages as a cell type contributing to disease pathogenesis and add additional consideration to the current T-cell-centric classification of iPALF. The fact that intrahepatic signatures were shared across different, but inflammation-related PALF etiologies may point to imperfections in our diagnostic pipelines, assay detection limits, or suggest that the destructive mechanisms in the liver may follow a downstream unified process.

### Whole-genome and exome sequencing techniques in PALF

At a surface level, iPALF does not have an immediately obvious monogenic or oligogenic cause. Genomic sequencing has been increasingly utilized in the diagnostic pipeline of iPALF secondary to the increase in ubiquity, affordability, and availability of these technologies in the clinical setting, particularly in tertiary pediatric medical centers. Whole-genome and exome sequencing (WGS/WES) has improved our diagnostic capability, and a significant proportion of iPALF cases are now being reclassified to their corresponding known etiology according to established deleterious genetic variants. Recently, a large multinational WES study from 19 countries included 260 cases of iPALF, largely outside of the neonatal period, where genetic causes are more prominent.^53^ Of 260 patients, 37% of cases had genetic variants with pathologic significance, spread across 36 ALF-associated genes.^53^ Consistent with traditional expectations, patients who present in their first year of life and those with recurrent episodes were the most likely to have a genetic cause identified. Of all identified mutations, 45% of the diagnosed cases fell within a mitochondrial disease group, followed by 28% with vesicular trafficking disorders, and 10% with cytosolic aminoacyl-tRNA synthetase deficiencies. *NBAS*, a gene responsible for ER trafficking, was the most prevalent causal gene.^53^

Notably, *NBAS* mutations can cause deleterious quantitative and qualitative effects to the immune system.^54^ NBAS-deficient patients were shown to have reductions in circulating CD56^dim^ NK and CD19+ naïve B-cell populations. Patients with *NBAS* mutations also had significantly decreased NK degranulation as measured by surface CD107a upon exposure to a target cell K562.^54^ It is theorized that these immune deficiencies predispose NBAS-deficient patients to febrile illnesses, which induce ER stress and activation of the integrated stress response, leading to cellular apoptosis and necrosis.^55^

## CONCLUSIONS

Although the last decade has seen great advancements in our understanding of the cellular mechanisms of iPALF, significant knowledge gaps remain. There is substantial evidence to support the concept that TC-PALF is a distinct phenomenon that is responsible for the majority of previously termed “iPALF” cases. However, the pathophysiologic description of TC-PALF remains incomplete. The exact mechanism of T-cell activation, the role of non-T-cell immune cells, and why TC-PALF appears to disproportionally affect the young are still large areas of speculation. Furthermore, the relationship between TC-PALF and cases of hepatitis/PALF associated with AAV viruses remains undefined. With the development and increased accessibility of precision “omic” techniques to investigate rare patient samples outside of the neonatal period, where genetics dominates, the potential to define the immune microenvironment of disease states with unprecedented granularity is possible. For children with TC-PALF, a comprehensive understanding of the disease pathogenesis may lead to biology-informed targeted immunotherapies, preventing the need for broad immunosuppression and ultimately, reducing the need for liver transplant.

Over the next decade, we are likely to see a rapid evolution in our understanding of immune-mediated diseases as advanced single-cell technology becomes more available. It is certain that with a greater ability to define cellular microenvironments, our appreciation for the complexity of immune cell interactions will grow. Current thinking around TC-PALF and other poorly understood immune diseases tends to focus on broad cell categories, such as macrophages and T cells. The first generation of single-cell studies has already proven that cellular identities are more complex than previously believed.^56^ The complexity of these disorders of immune dysregulation may be daunting, but our growing cumulative knowledge has the potential to transform our ability to diagnose and treat our patients.
